# The ProtecT randomised trial cost-effectiveness analysis comparing active monitoring, surgery, or radiotherapy for prostate cancer

**DOI:** 10.1038/s41416-020-0978-4

**Published:** 2020-07-16

**Authors:** Sian M. Noble, Kirsty Garfield, J. Athene Lane, Chris Metcalfe, Michael Davis, Eleanor I. Walsh, Richard M. Martin, Emma L. Turner, Tim J. Peters, Joanna C. Thorn, Malcolm Mason, Prasad Bollina, James W. F. Catto, Alan Doherty, Vincent Gnanapragasam, Owen Hughes, Roger Kockelbergh, Howard Kynaston, Alan Paul, Edgar Paez, Derek J. Rosario, Edward Rowe, Jon Oxley, John Staffurth, David E. Neal, Freddie C. Hamdy, Jenny L. Donovan

**Affiliations:** 1 Bristol Medical School, University of Bristol, Bristol, UK; 2 Bristol Randomised Trials Collaboration, Bristol Trials Centre, University of Bristol, Bristol, UK; 3 National Institute for Health Research, Bristol Biomedical Research Centre, University of Bristol, Bristol, UK; 4 The School of Medicine, University of Cardiff, Cardiff, UK; 5 Department of Urology and Surgery, Western General Hospital, Edinburgh, UK; 6 The Academic Urology Unit, University of Sheffield, Sheffield, UK; 7 Department of Urology, Queen Elizabeth Hospital, Birmingham, UK; 8 The Academic Urology Group, University of Cambridge, Cambridge, UK; 9 Cambridge Urology Translational Research and Clinical Trials Office, Cambridge, UK; 10 Department of Urology, Cardiff and Vale University Health Board, Cardiff, UK; 11 Department of Urology, University Hospitals Leicester, Leicester, UK; 12 Department of Urology, Leeds Teaching Hospitals NHS Trust, Leeds, UK; 13 Department of Urology, Freeman Hospital, Newcastle-upon-Tyne, UK; 14 Department of Urology, Sheffield Teaching Hospitals, Sheffield, UK; 15 Bristol Urological Institute, North Bristol NHS Trust, Bristol, UK; 16 Department of Cellular Pathology, North Bristol NHS Trust, Bristol, UK; 17 Nuffield Department of Surgical Sciences, University of Oxford, Oxford, UK

**Keywords:** Prostate cancer, Health care economics

## Abstract

**Background:**

There is limited evidence relating to the cost-effectiveness of treatments for localised prostate cancer.

**Methods:**

The cost-effectiveness of active monitoring, surgery, and radiotherapy was evaluated within the Prostate Testing for Cancer and Treatment (ProtecT) randomised controlled trial from a UK NHS perspective at 10 years’ median follow-up. Prostate cancer resource-use collected from hospital records and trial participants was valued using UK reference-costs. QALYs (quality-adjusted-life-years) were calculated from patient-reported EQ-5D-3L measurements. Adjusted mean costs, QALYs, and incremental cost-effectiveness ratios were calculated; cost-effectiveness acceptability curves and sensitivity analyses addressed uncertainty; subgroup analyses considered age and disease-risk.

**Results:**

Adjusted mean QALYs were similar between groups: 6.89 (active monitoring), 7.09 (radiotherapy), and 6.91 (surgery). Active monitoring had lower adjusted mean costs (£5913) than radiotherapy (£7361) and surgery (£7519). Radiotherapy was the most likely (58% probability) cost-effective option at the UK NICE willingness-to-pay threshold (£20,000 per QALY). Subgroup analyses confirmed radiotherapy was cost-effective for older men and intermediate/high-risk disease groups; active monitoring was more likely to be the cost-effective option for younger men and low-risk groups.

**Conclusions:**

Longer follow-up and modelling are required to determine the most cost-effective treatment for localised prostate cancer over a man’s lifetime.

**Trial registration:**

Current Controlled Trials number, ISRCTN20141297: http://isrctn.org (14/10/2002); ClinicalTrials.gov number, NCT02044172: http://www.clinicaltrials.gov (23/01/2014).

## Background

Treatments recommended for cancer localised within the prostate gland include radical surgery, radiotherapy, and active monitoring/surveillance, where radical treatment is avoided or delayed unless/until the cancer shows signs of progression. The ProtecT randomised treatment trial showed there was no evidence of a difference in prostate cancer mortality at a median of 10 years’ follow-up between 3D-conformal radiotherapy with neo-adjuvant androgen deprivation therapy (ADT), radical surgery, and active monitoring in men with clinically localised prostate cancer.^1^ Men randomised to surgery and radiotherapy had half the rate of prostate cancer progression and metastasis compared with the active monitoring group, but they experienced greater levels of treatment side effects, including urinary incontinence, erectile dysfunction and bowel symptoms.^2^

In most high- and middle-income countries, healthcare priorities are considered in the context of treatment effectiveness and its cost. Of two trials that compared surgery with watchful waiting,^3,4^ only the Scandinavian Prostate Cancer Group-4 trial (SPCG-4), conducted in the pre-PSA (Prostate Specific Antigen) era, evaluated costs. SPCG-4 found costs 34% higher for surgery compared with watchful waiting at a median of 12 years’ follow-up. The higher costs for surgery related to the initial treatment costs.^3^ In the absence of more contemporary trial data, models have been developed to estimate cost-effectiveness of treatments but have produced conflicting results.^5–9^

This paper presents an individual patient data economic evaluation of the ProtecT trial in terms of costs to the UK NHS and Quality-Adjusted-Life-Years (QALYs) at a median of 10 years’ follow-up, the prespecified time point for the primary analysis.^1^

## Methods

### Study design and participants

The ProtecT trial’s design and protocol have been published elsewhere.^1,10^ In brief, men in nine UK centres who were detected and diagnosed with clinically localised prostate cancer following a programme of population-based PSA testing who met trial eligibility criteria were randomised to active monitoring (*n* = 545), surgery (*n* = 553), or radiotherapy with neo-adjuvant ADT (*n* = 545).

### Resource-use data collection, coding, and valuation

A UK NHS perspective was used for the study; hence, health service resource-use data related to prostate cancer and treatments were recorded from randomisation until November 2015 (a median of 10 years’ follow-up). Resource-use relating to initial treatments and subsequent interventions were recorded onto study-specific proformas. Follow-up inpatient stays, outpatient, Emergency Department and, from April 2005, primary care attendances were recorded onto schedules at annual research nurse appointments with participants and from a hospital medical records review. To ensure data completeness, treatment proformas and annual schedules were compared, duplicate data dropped, and combined sources of data used. The trial database containing some clinical information was used for validation purposes. Supplementary Table 1 outlines how the resources were measured, coded and valued (2014–2015 UK £ prices) and Supplementary Table 2 outlines how missing resource-use and EQ-5D-3L (QALY) data were handled.

### Outcome measurement

The outcome for the economic analysis is the QALY at a median of 10 years’ follow-up. As recommended by NICE, utility values were estimated from the Euroqol EQ-5D-3L questionnaire and associated societal UK utility tariffs.^11^ The EQ-5D-3L was participant-completed prior to diagnosis (baseline), at 6 months, and annually from each man’s randomisation date. Men who died were assigned a zero-utility value for the remaining years they could have been trial participants. Utility values were combined to estimate the number of QALYs for each participant using the area-under-the-curve approach.^11^

### Analysis

The analysis compared the three groups as randomised, considering prostate cancer-related NHS costs in relation to QALYs for a median of 10 years. Costs and outcomes for the 10-year median analysis were discounted at 3.5%.^12^ Analyses were conducted in Stata 14.1.^13^ Each item of resource used was summed for each man and the mean resource-use calculated by category (e.g. inpatient stays for infection) and trial group, over the median 10-year period. Each item’s cost was calculated as the use (e.g. number of GP visits) multiplied by its unit cost and were summed annually, across time and by resource-use category for each participant.

Annual adjusted mean costs and QALYs were estimated using linear regression. The method of ‘seemingly unrelated regressions’ (SUR), which accounts for the correlation between costs and QALYs, was used to estimate total adjusted mean costs and QALYs.^14^ Costs and QALYs were adjusted for study centre, age, Gleason score (<7, 7, 8–10) and PSA at baseline, in keeping with the primary outcome analysis. QALYs were also adjusted for baseline utility.^11^ Across the three treatment groups, the adjusted mean costs and QALYs were compared to assess if any of the treatments were less effective and more expensive than the other treatments. If that was the case, then incremental cost-effectiveness ratios (ICERs) would not be estimated in relation to that treatment.^15^ Incremental adjusted mean costs and QALYs, bias-corrected and accelerated confidence intervals (to account for non-normal distributions), and ICERs were estimated using SUR and non-parametric bootstrapping (5000 model iterations). Regression outputs were used to estimate parametrically the incremental net monetary benefit (iNMB) statistic and associated confidence intervals at the UK NICE willingness-to-pay threshold of £20,000 per QALY.^12^

Cost-effectiveness acceptability curves (CEACs) were generated to explore sampling uncertainty in the cost-effectiveness estimates,^16^ presenting the probability that each trial group is the cost-effective option compared to the other two groups at a range of monetary values. To calculate the CEACs, individual net monetary benefit values were calculated at each willingness to pay per QALY threshold (£0–£100,000 at £1000 intervals). At each threshold, 5000 bootstrap model iterations of the adjusted linear regression models of the net monetary benefits were performed. The proportion of times (that is the probability) that each treatment group had the highest net monetary benefit at each threshold was then calculated and plotted to create the CEAC. One-way and scenario sensitivity analyses were used to account for methodological uncertainty or assumptions made during the study and analysis (see Supplementary Information). Exploratory subgroup analyses explored heterogeneity within the study population for age (<65 vs. 65+ years at randomisation); Grade group (1 vs. 2 and higher; and D’Amico risk classification (low vs. intermediate and high).^17^ These were chosen to reflect more relevant contemporary classifications of the prespecified subgroups in the ProtecT primary analysis.^1^

## Results

The adjusted cost and QALY SUR analysis were based on 1101 (67%) of the 1643 men randomised into the ProtecT trial. More data were available for other analyses (see Table 1, Supplementary Tables 3–4 for sample sizes).

**Table 1:** Total unadjusted mean resource-use and cost by allocation arm.

	Active monitoring (*n* = 513)^a^		Radiotherapy (*n* = 516)^a^		Radical prostatectomy (*n* = 527)^a^	
	Mean number of units^b^	Mean cost (£) (SD)	Mean number of units^b^	Mean cost (£) (SD)	Mean number of units^b^	Mean cost (£) (SD)
Hospital outpatients visits						
Procedure-driven appointments						
Protocol radiotherapy	7.77	846 (1608)	28.37	3090 (1665)	3.69	402 (1190)
Non-protocol radiotherapy	1.01	133 (631)	0.37	49 (377)	1.86	245 (835)
CT scan	0.29	26 (53)	0.88	81 (56)	0.22	20 (52)
Radiotherapy preparation	0.27	169 (279)	0.80	503 (252)	0.19	122 (249)
TRUS	0.35	19 (33)	0.29	16 (30)	0.29	16 (30)
Trial without a catheter	0.17	24 (57)	0.09	13 (43)	0.53	73 (73)
Bone scan	0.28	56 (176)	0.11	23 (97)	0.13	26 (102)
MRI scan	0.20	27 (59)	0.13	17 (53)	0.11	15 (45)
TRUS-guided biopsy	0.16	35 (83)	0.07	15 (113)	0.04	9 (47)
Chemotherapy	0.04	58 (663)	0.09	145 (1570)	0.03	50 (844)
Other procedures	0.50	60 (250)	0.32	57 (346)	0.34	55 (569)
Speciality-driven appointments						
Urology	8.99	868 (787)	9.56	913 (795)	11.64	1122 (810)
Oncology	1.08	156 (463)	2.76	400 (646)	1.31	190 (536)
Uro-oncology	0.62	80 (205)	1.07	137 (235)	0.80	102 (199)
Other specialities^c^	16.56	912 (519)	4.79	374 (577)	3.98	260 (493)
Total outpatient cost		3469 (2754)		5832 (3106)		2707 (2748)
Hospital day case stays						
Flexible cystoscopy	0.11	51 (165)	0.06	29 (119)	0.08	35 (142)
Colonoscopy	0.04	21 (126)	0.07	34 (185)	0.02	8 (63)
TWOC	0.04	16 (83)	0.01	2 (29)	0.05	19 (92)
Sigmoidoscopy	0.01	5 (46)	0.04	19 (100)	0.02	7 (81)
Rigid cystoscopy	0.03	27 (160)	0.01	10 (107)	0.04	33 (189)
TRUS-guided biopsy	0.05	25 (117)	0.01	5 (52)	0.01	4 (46)
Education	0.02	10 (70)	0.01	4 (52)	0.02	9 (75)
Chemotherapy	0.01	13 (285)	0.01	21 (390)	0.03	57 (936)
Urodynamics	0.01	2 (25)	0.01	2 (25)	0.02	6 (43)
Brachytherapy	0.01	10 (97)	0.01	6 (75)	0.01	6 (75)
Other day case reasons	0.14	99 (427)	0.12	81 (417)	0.16	119 (505)
Total day case cost		277 (689)		214 (715)		303 (1299)
Hospital inpatient stays						
Prostatectomy	0.25	1332 (2380)	0.11	578 (1709)	0.77	4013 (2347)
Infection	0.02	31 (278)	0.02	42 (721)	0.04	76 (509)
TURP	0.05	142 (667)	0.02	44 (333)	0.00	21 (379)
Brachytherapy	0.03	61 (347)	0.02	32 (240)	0.02	31 (237)
Bladder neck procedure	0.01	18 (213)	0.01	30 (278)	0.02	44 (443)
Insertion of urinary sphincter	0.01	129 (1238)	0.01	70 (796)	0.02	206 (1464)
Pain	0.01	17 (243)	0.01	10 (115)	0.02	28 (323)
Urinary retention	0.01	17 (143)	0.01	7 (94)	0.01	14 (131)
Rigid cystoscopy	0.00	3 (60)	0.01	19 (158)	0.01	16 (168)
Blood transfusion	0.02	18 (255)	0.00	0 (0)	0.00	4 (90)
Other inpatient stays	0.15	434 (2298)	0.08	235 (1458)	0.18	403 (2316)
Total inpatient cost		2202 (3956)		1068 (2854)		4855 (4055)
GP practice visits						
By healthcare professional						
GP	0.81	36 (79)	0.93	41 (86)	1.09	48 (92)
GP nurse	0.91	13 (38)	0.66	10 (34)	0.69	10 (36)
Other	0.05	1 (5)	0.07	1 (9)	0.12	2 (16)
By reason						
PSA test	27.41	435 (142)	20.91	332 (140)	20.80	330 (138)
Hormone delivery	1.97	44 (146)	4.24	94 (87)	0.85	19 (87)
Total GP practice cost		528 (243)		477 (205)		409 (231)
Medications						
Cyproterone acetate days^d^	0.23	1 (5)	0.37	1 (7)	0.16	0 (4)
Hormone injections	2.48	277 (770)	3.96	331 (515)	1.20	154 (606)
Total medication cost		277 (770)		332 (515)		154 (606)
Total cost		6754 (5597)		7923 (4717)		8428 (5636)

^a^Given the missing data assumptions (Supplementary Table 2), resource-use was obtained for 1556 (95%) of the 1653 men randomised into the ProtecT study.

^b^Units refer as appropriate to: number of outpatient appointments; number of day case visits; number of inpatient stays; number of GP practice visits; number of medications; number of days.

^c^Includes Accident and Emergency visits that did not lead to a procedure costed by the HRG.

^d^Assuming a daily dose of 200 mg.

### Resource-use

Resources related to primary treatments varied by randomised groups (Table 1), with the radiotherapy group having more outpatient visits, colonoscopy and sigmoidoscopy procedures, and fewer inpatient stays. There were more primary care resources, biopsies, magnetic resonance imaging (MRI) scans, bone scans, and transurethral resections of the prostate (TURP) in the active monitoring group, and more infection and urinary sphincter-related inpatient stays in the surgery group.

### Basecase results

**Fig. 1:**
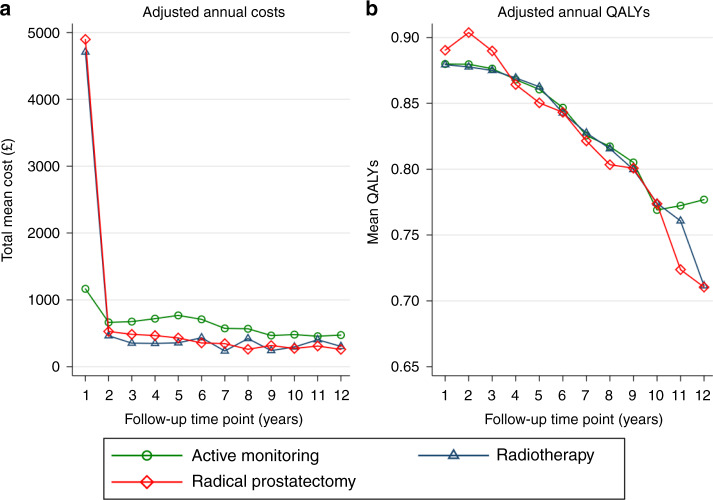
Line charts displaying for each annual follow-up time point, by allocation arm (Active monitoring, Radiotherapy, Radical prostatectomy) the (**a**) mean adjusted annual costs and (**b**) mean adjusted annual QALYs (see Supplementary Tables 3 and 4 for sample sizes).

Over the median 10 years’ follow-up, the total adjusted mean costs of the surgery (£7519: 95% CI £7099–£7940) and radiotherapy (£7361: 95% CI £6938–£7783) groups remained very similar. Both radical groups were more expensive than the active monitoring group (£5913: 95% CI £5494–£6332). Year 1 adjusted mean costs were also similar for surgery (£4898) and radiotherapy (£4708), but much lower for active monitoring (£1166 (Supplementary Table 3; Fig. 1)).

The total adjusted mean QALYs were similar for all groups—just slightly higher for those allocated to radiotherapy (7.093 QALYs: 95% CI 6.914–7.273) compared with active monitoring (6.976 QALYs: 95% CI 6.798–7.154) and surgery (6.909 QALYs: 95% CI 6.731–7.087) (Table 2).

**Table 2:** Cost-effectiveness results.

Allocation arm	*n*^a^	Adjusted^b^ costs (£) Mean (95% CI)	Adjusted^b^ QALYs Mean (95% CI)	Comparison	Incremental cost (£) (95% CI)^c^	Incremental QALY (95% CI)^d^	ICER (£/QALY)^d^	Incremental NMB (£) at £20,000/ QALY (95% CI)
Active monitoring (AM)	370	5913 (5494–6332)	6.976 (6.798–7.154)					
Radiotherapy (RT)	364	7361 (6938–7783)	7.093 (6.914–7.273)	RT vs. AM	1448 (803–2061)	0.118 (−0.141 to 0.368)	12,310	904 (−4181 to 5990)
Radical prostatectomy (RP)	367	7519 (7099–7940)	6.909 (6.731–7.087)	RP vs. RT	159 (−410 to 747)	−0.184 (−0.431 to 0.073)	RT dominates^e^ RP	−3847 (−8940 to 1245)

^a^Including only 1101 participants for whom we have complete cost and QALY information.

^b^Adjusted for the minimisation variables of the randomisation process: study centre, age at baseline, Gleason score (2–6, 7, 8–10) and PSA at baseline. QALYs were also adjusted for baseline utility.

^c^Bias-corrected and accelerated confidence interval based on 5000 bootstrap replications.

^d^The ICER cannot be estimated directly from the incremental costs and QALYs due to rounding.

^e^Dominates means that the treatment is less costly and more effective than the other treatment.

**Fig. 2:**
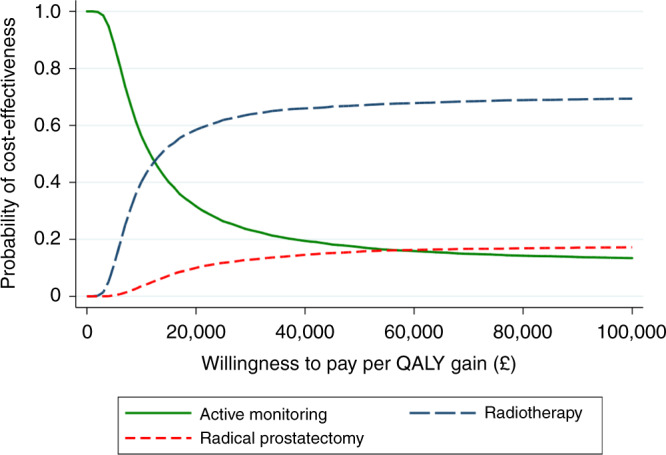
Cost-effectiveness acceptability curve displaying the probability of each treatment (Active monitoring, Radiotherapy, Radical prostatectomy) being the cost-effective option at different willingness-to-pay thresholds.

The result that the radiotherapy group was more expensive than the active monitoring group but had slightly higher QALYs meant that it was appropriate to estimate an ICER. The ICER of £12,310 per QALY showed that radiotherapy was the cost-effective option at the standard UK NICE willingness-to-pay threshold of £20,000 per QALY. The CEAC (Fig. 2), reflecting sample uncertainty, shows at this threshold the probability that radiotherapy is the cost-effective option is 58%, with a probability of 32% for active monitoring and 10% for surgery.

### Sensitivity analyses

Sensitivity analyses (Supplementary Table 5) confirmed these results for the most part, except when QALY data were not imputed, or when only including participants following the introduction of primary care data collection, or excluding men recruited during the feasibility period; and the scenario using newer techniques (robotic surgery and intensity-modulated radiation therapy (IMRT)). In these four analyses, radiotherapy would be cost-effective at the higher UK-NICE willingness-to-pay threshold of £30,000 per QALY.

### Subgroup analyses

Subgroup analyses (Table 3) showed that QALYs were again similar between groups, but costs varied. For example, for younger men and those with lower risk disease (D’Amico classification), active monitoring was less costly and had higher QALYs than the radical groups and so was most likely to be the cost-effective option. However, for older men and those with higher risk disease, radiotherapy was most likely to be the cost-effective option. At the £20,000 per QALY threshold, the probability that active monitoring was the cost-effective option was 80% for younger men, 84% for D’Amico low-risk disease, and 66% for Grade group 1; and that radiotherapy was the cost-effective option was 73% for older men, 69% for D’Amico intermediate/high-risk disease, and 75% for Grade group 2 or higher.

**Table 3:** Subgroup cost-effectiveness results.

Allocation arm	*n*^a^	Adjusted costs (£) Mean (95% CI)	Adjusted QALYs Mean (95% CI)	Comparison	Incremental cost (£) (95% CI)^b^	Incremental QALY (95% CI)^b^	ICER^c^ (£/QALY)	Incremental NMB (£) at £20,000/ QALY (95% CI)
Age group 1: Younger—under 65 years old at randomisation^d^								
Active monitoring (AM)	229	6030 (5534−6526)	7.175 (6.945−7.404)					
Radical prostatectomy (RP)	232	7458 (6966−7950)	6.933 (6.706−7.160)	RP vs. AM	1428 (759−2114)	−0.241 (−0.571 to 0.085)	AM dominates^e^ RP	−6257 (−12,763 to 250)
Radiotherapy (RT)	223	7585 (7083−8087)	7.095 (6.863−7.327)	RT vs. AM	1555 (841−2345)	−0.079 (−0.417 to 0.241)	AM dominates RT	−3142 (−9719 to 3435)
Age group 2: Older—65 years old and over at randomisation^d^								
Active monitoring (AM)	141	5671 (4927−6415)	6.654 (6.376−6.933)					
Radiotherapy (RT)	141	7047 (6304−7789)	7.104 (6.825−7.382)	RT vs. AM	1376 (134−2312)	0.449 (0.045−0.875)	3061	7612 (−337 to 15,561)
Radical prostatectomy (RP)	135	7637 (6874−8400)	6.853 (6.568−7.138)	RP vs. RT	590 (−349 to 1754)	−0.250 (−0.621 to 0.144)	RT dominates RP	−5599 (−13,665 to 2467)
D’Amico group 1: Low^f^								
Active monitoring (AM)	217	4833 (4359−5308)	7.060 (6.830−7.290)					
Radical prostatectomy (RP)	226	6806 (6344−7268)	6.843 (6.619−7.067)	RP vs. AM	1973 (1272−2620)	−0.217 (−0.551 to 0.103)	AM dominates RP	−6314 (−12,772 to 144)
Radiotherapy (RT)	228	7050 (6585−7514)	7.017 (6.792−7.242)	RT vs. AM	2216 (1512−2931)	−0.043 (−0.381 to 0.267)	AM dominates RT	−3078 (−9566 to 3410)
D’Amico group 2: Intermediate/high^f,g^								
Active monitoring (AM)	120	7453 (6548−8358)	6.494 (6.194−6.794)					
Radiotherapy (RT)	110	7852 (6909−8795)	6.874 (6.561−7.188)	RT vs. AM	399 (−1086 to 1560)	0.380 (−0.049 to 0.849)	1049	7209 (−1604 to 16,023)
Radical prostatectomy (RP)	108	8807 (7852−9762)	6.668 (6.352−6.985)	RP vs. RT	955 (−267 to 2246)	−0.206 (−0.635 to 0.228)	RT dominates RP	−5079 (−14,131 to 3972)
Grade group 1^h^								
Active monitoring (AM)	275	5287 (4867−5708)	7.132 (6.924−7.340)					
Radical prostatectomy (RP)	275	6892 (6472−7312)	6.997 (6.790−7.205)	RP vs. AM	1605 (999−2223)	−0.135 (−0.453 to 0.163)	AM dominates RP	−4302 (−10,233 to 1629)
Radiotherapy (RT)	279	7029 (6612−7446)	7.144 (6.938−7.350)	RT vs. AM	1742 (1143−2388)	0.012 (−0.288 to 0.303)	146,076	−1503 (−7413 to 4407)
Grade group 2 and higher^h,i^								
Active monitoring (AM)	95	7799 (6694−8904)	6.558 (6.223−6.892)					
Radiotherapy (RT)	85	8522 (7359−9685)	6.943 (6.591−7.295)	RT vs. AM	723 (−1160 to 2337)	0.385 (−0.108 to 0.905)	1878	6974 (−2819 to 16,767)
Radical prostatectomy (RP)	92	9249 (8130−10,369)	6.598 (6.259−6.937)	RP vs. RT	727 (−856 to 2188)	−0.345 (−0.797 to 0.132)	RT dominates RP	−7,625 (−17,445 to 2194)

^a^Including only participants for whom we have complete cost and QALY information.

^b^Bias-corrected and accelerated confidence interval based on 5000 bootstrap replications.

^c^ICERs cannot be estimated directly from the incremental costs and QALYs due to rounding.

^d^Adjusted for study centre, Gleason score (2–6, 7, 8–10) and PSA at baseline. QALYs were also adjusted for baseline utility.

^e^Dominates means that the treatment is less costly and more effective than the other treatment.

^f^Adjusted for study centre and age at baseline. QALYs were also adjusted for baseline utility.

^g^ D’Amico group 2, 0.02% of bootstrap replicates failed to converge.

^h^Adjusted for study centre, age at baseline, and PSA at baseline. QALYs were also adjusted for baseline utility.

^i^Grade group 2 and higher, 0.7% of bootstrap replicates failed to converge.

## Discussion

In this cost-effectiveness analysis of the ProtecT treatment trial, there were remarkably small differences between the treatment groups at a median of 10 years. Overall, radiotherapy had the greatest probability of being the cost-effective option for localised prostate cancer at a median of 10 years follow-up at the UK NICE willingness-to-pay threshold of £20,000 per QALY and was most likely to be the cost-effective option at all higher thresholds.

The marginally higher QALYs in the radiotherapy group meant that although it was more costly than the active monitoring group, it was more likely to be the cost-effective option. While the subgroup analyses for both the higher risk, and 65 years and older groups mirrored these results with greater certainty, for younger men and/or those with low-risk disease (defined by the D’Amico classification) active monitoring was more likely to be the cost-effective treatment group.

Over the median 10-year period, the mean cost-difference between the radiotherapy and surgery groups was only £159. The higher surgery group cost was robust to all but the sensitivity analysis that costed robotic surgery and intensity-modulated radiotherapy, and in the subgroup analyses it was lower for younger men and the low-risk groups (range: £127–£244). Throughout all the analyses the surgery group had fewer QALYs than the radiotherapy group; the difference in QALYs would translate into a reduction of 67 days in the best imaginable health over a median of 10 years.

The finding that the active monitoring group had the lowest costs may address clinician concern that active surveillance could be more expensive in the long term because of the need to keep monitoring patients who might ultimately end up having treatment. This study shows that at a median 10 years this was not the case.

The uncertainty reflected in only a 58% probability that the radiotherapy group was the cost-effective option needs to be acknowledged; the result indicates that it is inconclusive as to which treatment over the median 10 years would be the best value for money. For older men and/or those with higher risk disease, there was more certainty that the radiotherapy group was the cost-effective option; similarly, there was more certainty that the active monitoring group was the cost-effective option for younger men and/or those with low-risk disease—although these findings, being from subgroup analyses were conducted on smaller numbers and therefore need to be interpreted with some caution.

This study is the first and only economic evaluation within a randomised trial comparing the three contemporary major treatment modalities (ProtecT). The only previous randomised evidence came from SPCG-4, comparing surgery with watchful waiting—not active monitoring, and in the pre-PSA era. This showed higher costs in the surgery arm.^3^ The findings from this study are discordant with some modelling studies. To facilitate comparisons, published costs from the modelling studies were inflated to 2015 prices^18^ and converted to relevant currencies using 2015 purchasing power parities.^19^ Two lifetime modelling studies based on 65-year-old men indicated that active surveillance was the most cost-effective strategy compared with radical prostatectomy (7.6 QALYs: €9585 (£9260 vs. 7.56 QALYs: €16,468 (£15,911)^8^ and 8.85 QALYs: US$39,894 (£28,796) vs. 7.95 QALYs: US$38,180 (£27,559)^6^) and IMRT (8.10 QALYs: US$48,699 (£35,152)^6^]. In two models using SPCG-4 data, surgery was shown to be cost-effective, although it was more expensive than watchful waiting for 65-year-old men (ICER: 58,045 SEK (£4957) per QALY)^20^ and when an active surveillance programme was considered (ICER: NZ$33,160 (£16,070)).^9^ The limited availability of outcome data has been acknowledged to have been a weakness of the models produced.^6,8^

The main strength of this study is that it is based on individual patient data from the ProtecT randomised trial comparing the three contemporary major treatment modalities over a 10-year median duration. The use of medical records in conjunction with a participant visit meant that hospital-based missing data were minimised and likely to be missing completely at random. The presentation of resources used and their costs, and the use of different willingness-to-pay per QALY thresholds, means that country-specific costs and thresholds can be applied to these data.

Limitations of the study include changes that have occurred during the study’s duration. New techniques such as robot-assisted surgery and IMRT have become more prevalent and are potentially more expensive; in addition, there has been a global shift to shorter treatment courses of radiotherapy. A sensitivity analysis in which surgery was costed as robot-assisted, and radiotherapy as intensity-modulated radiotherapy meant that the radiotherapy group was only cost-effective at the higher £30,000 per QALY threshold; however, reducing the number of fractions delivered led to the radiotherapy arm becoming more cost-effective. Active surveillance programmes have also changed over time. Improved diagnostic techniques (mpMRI) might enable better selection of patients, which could lead to fewer changing to radical treatments or developing metastases, potentially lowering costs and improving effectiveness.

Another important limitation relates to the small number of events in the ProtecT trial to date because of the long natural history of prostate cancer, meaning that longer follow-up is needed to establish whether there are differences in mortality between the groups—which could change the balance of cost-effectiveness. In the late 1990s when the ProtecT trial was being designed, a 10-year time horizon was expected to show differences in clinical events. The exploratory subgroup analyses suggested differences in costs and effectiveness between age and prostate cancer risk groups. It would thus be premature to imply ruling out any of the treatment options at the present time, particularly following the 23-year follow-up of the SPCG-4 trial demonstrating improved survival in men receiving surgery compared with watchful waiting.^21^ Longer-term follow-up is needed in ProtecT to ascertain survival differences.

Other limitations relate to data collection and analysis. Primary care resource-use was not recorded in the follow-up schedules at the beginning of the trial. Therefore, all neo-adjuvant ADT treatment and PSA tests recorded in the trial database were costed. A sensitivity analysis including only participants with all primary care visits recorded indicated that the radiotherapy group was only cost-effective at the higher £30,000 per QALY threshold. Missing data occurring within the follow-up proformae were imputed following discussions with clinical staff, and a researcher blinded to treatment allocation removed duplicated events between sources, which could have led to small errors. Incomplete EQ-5D-3L data meant QALYs could only be calculated for 69% of the sample and could have been missing not at random, for example due to ill health. There is a suggestion that participants missing from the surgery group may have been less healthy at baseline than those missing in the other two arms (Supplementary Table 6), potentially leading to an overestimate in the mean QALY reported for the surgery group, but not changing the overall findings.

## Conclusion

In the primary economic analysis at a median of 10 years, radiotherapy was the most likely cost-effective option because of slightly lower initial costs and slightly more QALYs, but this result was not conclusive. In subgroup analyses, there was more certainty that radiotherapy was the cost-effective option for older men and/or those with intermediate/high-risk disease. Active monitoring was the least costly option overall, and subgroup analyses suggested it was the most likely cost-effective option for younger men and/or those with low-risk disease. This evaluation provided evidence of the cost-effectiveness of the major primary treatment modalities for clinically localised prostate cancer over a median 10-year period. Further follow-up and subsequent modelling are required to compare other types of treatments, including different radiotherapy modalities, and to assess the impact of later stages of progressing disease to establish which treatment might be most cost-effective over a man’s lifetime. In the meantime, all treatments should continue to be offered, with patients, clinicians, and policy-makers using these results in combination with the evidence from ProtecT in relation to the trade-offs between disease progression, metastases, and urinary, sexual and bowel function.

## Supplementary information

supplementary information

## Data Availability

On request to the ProtecT study, we will provide a patient deidentified set of EQ-5D-3L scores from Baseline (Biopsy) onwards, mortality information and annual costs at the Outpatient, Inpatient, GP and medication level, for prostate cancer-related research as per informed consent for researchers within the EU.
