# Supplementary material for: The ProtecT randomised trial cost-effectiveness analysis comparing active monitoring, surgery, or radiotherapy for prostate cancer
Source: Br J Cancer. 2020 Jul 16;123(7):1063–70. doi: 10.1038/s41416-020-0978-4 (PMC7524753; doi:10.1038/s41416-020-0978-4)
Supplement: Supplementary file 1 — supplementary information [file 41416_2020_978_MOESM1_ESM.docx]

Supplementary Information

The ProtecT randomised trial cost-effectiveness analysis comparing Active Monitoring, Surgery, or Radiotherapy for Prostate Cancer.

.

Table of contents

Changes from the original protocol Page 2

One-way sensitivity analyses Page 2

Scenario sensitivity analyses Page 3

Supplementary Table 1: Resource-use measurement, coding and valuation Page 4

Supplementary Table 2: Missing data assumptions Page 7

Supplementary Table 3: Mean adjusted costs per year, by arm Page 8

Supplementary Table 4: Mean adjusted QALYs per year, by arm Page 9

Supplementary Table 5: Results of the one-way and scenario sensitivity analyses Page 10

Supplementary Table 6: Baseline characteristics of those included and excluded from the

SUR analysis by arm Page 15

References Page 16

**Changes from the original Protocol**

The economic evaluation section of the ProtecT protocol was written early in the trial, the main differences between the protocol analysis and the actual analysis are as follows:

1)An NHS rather than a societal perspective was reported as a basecase analysis. NICE advocates that an NHS and personal social service perspective is used^1^. Information on patient costs and personal social services which were mainly obtained through two cross sectional surveys of the participants will be reported in a separate paper.

2) A 10-year median analysis was conducted rather than the 1, 5, 10 and 15 years specified in the protocol, as decision makers are interested in long term cost-effectiveness, and a 10-year median analysis is the longest time for which a within trial analysis can be performed.

3)A discount rate of 6% was specified in the protocol. The current discount rate of 3.5% was used^1^.

**One-way sensitivity analyses**

The first four sensitivity analyses were conducted to explore the effect of missing data:

1) The imputed mean QALY for those who had one timepoint missing was replaced with a QALY score 10% lower than this mean score to take into account the possibility that non-completion of questionnaires resulted from illness and treatment.

2) An analysis excluding the cases where the QALY had been imputed.

3) To examine the impact of not capturing information on primary care visits until 2005, the analysis was rerun for those participants who received their first annual follow-up following this date.

4) In order to examine whether the use of a median 10-year rather than a 10-year follow-up period and the resulting administrative censoring, may have affected the results, the analysis was rerun on the first 6 years of follow-up, the time point where no administrative censoring had occurred. The analysis was run again on the first 6 years adjusting for the information appointment date. The results of these two analyses were compared.

5) During the feasibility period of the study, initial follow-up was not as robust as for the main trial, the men from this period were therefore excluded from this sensitivity analysis.

6) The appropriate discount rate for economic evaluations in health care is the subject of debate internationally and varies between jurisdictions. For this reason, the impact of a 1.5% discount rate on both costs and benefits was explored.

7) It was sometimes difficult to distinguish between a daycase and outpatient procedure. All day cases were therefore costed as outpatient procedures in the sensitivity analysis.

8) Prostate cancer related resource-use was used for this analysis; however, unrelated resource use was also recorded. There was some discrepancy over whether a Trans Urethral Resection of the Prostate (TURP) was or not related to the active monitoring treatment. The increased surveillance of these men could have meant that more lower urinary tract symptoms (LUTS) may have been identified which could have meant more TURPs were performed. In the base case analysis all TURPS were included, but they were excluded from this sensitivity analysis.

9) In economic evaluations, research based appointments are not usually costed. Following discussion with the trial research nurses, it was discovered that clinical follow-up also occurred within annual research follow-up appointments. In the basecase analysis these appointments were costed as half an outpatient visit. In the sensitivity analysis these appointments were excluded.

10) There has been a global shift to shorter treatment courses in the delivery of radiotherapy. This sensitivity analysis alters the number of fractions given from 37 to 20.

11) The trial research nurses were asked to record all non-admission secondary care attendances in one section of the proforma. It was discovered through discussion with the nurses, that some nurses at certain times only recorded clinician follow-up in that section, and any outpatient procedures were recorded elsewhere in the proforma. Although the main procedures e.g. bone cans, Biopsies, MRI’s CT scans were accounted for, other procedures e.g. cystoscopy may not have been included by some nurses in the outpatient section and were not included in the basecase analysis. This sensitivity analysis therefore includes the costs of all other procedures recorded elsewhere in the proforma.

**Scenario sensitivity analyses**

1) A high cost option whereby: All Trial without catheters were costed as Day cases; An outpatient visit cost was assigned for extracting blood for psa tests; All procedures recorded only in the follow-up schedules were costed as the highest CC (complications and comorbidities split); Annual research follow-up appointments were costed as an outpatient visit and Initial and salvage radiotherapy planning were costed using technical support.

2) New techniques initial treatment option whereby: Radiotherapy was costed as Intensity-modulated radiation therapy (IMRT), which included planning, fiducial markers, image guided radiation therapy and the insertion of a rectal hydrogel spacer, and Radical Prostatectomy was costed as a Robotic Radical Prostatectomy

**Supplementary Table 1: Resource-use measurement, coding and valuation**

| **Resource** | **How it was measured** | **Assumptions made in relation to prevaluation coding and/or costing.** | **How it was valued** |
| --- | --- | --- | --- |
| Radical Prostatectomy | Number of each type of procedure | Standard open prostatectomies were assigned a Health Resource Group (HRG)4 code LB21A or LB21B dependant on complications and comorbidities, human key-hole prostatectomies were assigned LB22Z and robotic key-hole prostatectomies were assigned LB69Z. | UK’s Department of Health’s (2014-2015) reference costs^a,2^ |
| Outpatient delivery of hormones | Number of appointments | For participants, where hormones were recorded but no method of delivery was given, the first depot was assigned a non-consultant led (WF01A) and clinical oncology outpatient speciality (800) code^b^ | UK’s Department of Health’s (2014-2015) reference costs^2^ |
| Primary Care delivery of hormones | Number of appointments | The proportions of injections recorded that were delivered by GPs (26%) and practice nurses (74%) was used to create a weighted average cost. | Cost of a GP appointment^c^  Cost of a practice nurse appointment^d,3^ |
| Hormones delivered in primary care | Number of injections | Zoladex injections were costed using 1 3.6mg pre-filled disposable injection for monthly injections and using 1 10.8mg pre-filled disposable injection for 3-monthly injections. Leuprolide injections were costed using 1 3.75mg pre-filled disposable injection for monthly injections and using 1 11.25mg pre-filled disposable injection for 3-monthly injections. | BNF Online (current price was valid for 2015 Dictionary of Medicines and Devices)^4^ |
| Pre treatment planning | Allocated to each radiotherapy treatment | The HRG4 code SC51Z was used for protocol radiotherapy and salvage radiotherapy | UK Department of Health’s (2014-2015) reference costs^2^ |
| Radiotherapy delivery | Number of fractions | The HRG4 code SC22Z was used for protocol radiotherapy and SC23Z for salvage radiotherapy | UK Department of Health’s (2014-2015) reference costs^2^ |

**Supplementary Table 1: Resource-use measurement, coding and valuation (continued)**

| **Resource** | **How it was measured** | **Assumptions made in relation to prevaluation coding and/or costing.** | How it was valued |
| --- | --- | --- | --- |
| Active monitoring visits | Number of visits | The average time of a face to face and telephone consultation were calculated from the active monitoring schedules. | Cost of a Nurse team leader per minute of face to face contact^d^ |
| Taking of blood for PSA | Number of psa tests | All psa tests recorded in the clinical centres databases were allocated a weighted GP/nurse visit average cost using the recorded proportion of blood taken by GPs (4.7%) and practice nurses, phlebotomists and other staff (95.3%) | Cost of a GP appointment^c^,^4^  Cost of practice nurse appointment^d^,^3^ |
| Other Inpatients stays and day cases (no overnight stay) | Number of each type of procedure | Reasons for admission were mapped to OPCS4 codes. The HRG4+ 2014/15 Reference Costs Grouper Code to Group v1.0 workbook^5^ was used to allocate the HRG codes^e^. Assumption of no CC (comorbidities or complications) was made where an HRG split was made on CC. | Non-elective and elective inpatient costs assigned for overnight stays; Daycase costs assigned otherwise UK Department of Health’s (2014-2015) reference costs^a^ |
| Study annual research/clinical follow-up visit | Number of visits | Half the cost of an outpatient visit | UK Department of Health’s (2014-2015) reference costs^2^ |
| Chemotherapy | Number of visits | Outpatient chemotherapy was valued using an assumption of a 21 day cycle of Docetaxel and a weighted average cost of chemotherapy delivery HRG4s (SB12Z, SB13Z, SB14Z) plus a weighted average cost of chemotherapy procurement HRG4s (SB05Z, SB06Z, SB07Z, SB08Z, SB09Z, SB010Z)  Inpatient/day case chemotherapy was valued assuming Docetaxel using a chemotherapy delivery HRG4 of SB12Z and chemotherapy procurement HRG4 of SB08Z. | UK Department of Health’s (2014-2015) reference costs^2^ |

**Supplementary Table 1: Resource-use measurement, coding and valuation (continued)**

| **Resource** | **How it was measured** | **Assumptions made in relation to prevaluation coding and/or costing.** | **How it was valued** |
| --- | --- | --- | --- |
| Other outpatient consultations | Number of outpatient consultations for each speciality |  | UK Department of Health’s (2014-2015) reference costs^2^ |
| Outpatient procedures | Number of each type of procedure | Procedures were assigned an HRG4 code. An average outpatient procedure HRG cost was created from all relevant speciality outpatient procedure HRG costs. | UK Department of Health’s (2014-2015) reference costs^2^ |
| Other primary care consultations | Number of each type of consultation |  | Cost of a GP appointment^c^  Cost of a practice nurse appointment^d,3^ |

^a^Nights beyond the respective HRG trim point (the length of stay at the third quartile plus 1.5-times the inter-quartile range) were costed as excess nights;

^b^Includes the cost of the hormone injections;

^c^ Includes direct care staff costs and qualification costs and based on a 11.7minute consultation;

^d^Includes qualification costs and based on a 15.5 minute consultation;

^e^ For all events which had OPCS4 codes with a hierarchy of less than 5 or for events for which there was not enough information in the text to map onto an HRG code, these were allocated on the basis of the diagnosis, which in most cases was that of prostate cancer (OPCS code=C61X).

**Supplementary Table 2: Missing data assumptions**

|  | **Assumptions made in relation to missing resource-use data and EQ-5D-3L values** |
| --- | --- |
| Delivery of hormones | For participants, where hormones were recorded but no method of delivery was given, the first injection was assumed to be delivered in outpatients. From the second injection onwards, the hormones were assumed to be delivered in primary care and the number of appointments was determined by the number of hormone injections. |
| Hormones delivered in primary care | If the medication name was missing from the radiotherapy schedule, Zoladex was assumed (most common) and if the frequency was missing monthly was assumed (from ProtecT protocol). Hormones from follow-up schedule data were costed as Zoladex injections. If there were more than 4 injections per annum a monthly injection regime was assumed, otherwise a 3-monthly injection regime was assumed. |
| Radiotherapy delivery | Where number of fractions was missing it was assumed participants who had protocol radiotherapy had protocol number of fractions (37 fractions). Participants who had salvage radiotherapy but a missing number of fractions following clinician opinion were assumed to have 20 fractions based on a move towards 4-week cycles |
| Inpatients stays, day cases (no overnight stay), outpatient procedures and consultations | In the admissions, consultations, procedures recorded, assumed following consultation with research nurses that no events occurred. If number of outpatient visits were missing, assumed one visit as most research nurses questioned believed this would have been the case. For all events in which there was not enough information in the text to map onto a Health Resource Group (HRG) code, these were allocated on the basis of the diagnosis. If type of inpatient admission was absent, it was assumed to be an elective admission. If type of outpatient speciality was missing, a urology speciality code was used |
| Missing EQ-5D-3L scores in the year prior to the year of their death | The preceding year’s EQ-5D-3L score was used if available |
| EQ-5D-3L score missing at a timepoint | The mean of the adjacent years’ values was used, where available, otherwise the timepoint was left as missing |

**Supplementary Table 3: Mean adjusted costs per year, by arm**

| **Year** | **Active monitoring** | | | **Radiotherapy** | | | **Radical prostatectomy** | | |
| --- | --- | --- | --- | --- | --- | --- | --- | --- | --- |
|  | **N^a^** | **Mean cost (£)^b^** | **(SE)** | **N^a^** | **Mean cost (£)^b^** | **(SE)** | **N^a^** | **Mean cost (£)^b^** | **(SE)** |
| 1 | 513 | 1166 | (98) | 516 | 4708 | (98) | 527 | 4898 | (97) |
| 2 | 513 | 664 | (47) | 516 | 463 | (47) | 527 | 528 | (46) |
| 3 | 513 | 675 | (52) | 516 | 353 | (51) | 527 | 485 | (51) |
| 4 | 513 | 719 | (65) | 516 | 349 | (64) | 527 | 466 | (64) |
| 5 | 513 | 768 | (61) | 516 | 357 | (61) | 527 | 431 | (61) |
| 6 | 513 | 709 | (78) | 516 | 434 | (78) | 527 | 356 | (77) |
| 7 | 510 | 574 | (47) | 514 | 235 | (47) | 521 | 346 | (47) |
| 8 | 445 | 568 | (66) | 451 | 421 | (66) | 450 | 261 | (66) |
| 9 | 356 | 467 | (55) | 363 | 241 | (55) | 362 | 318 | (55) |
| 10 | 273 | 481 | (63) | 270 | 295 | (63) | 279 | 273 | (62) |
| 11 | 180 | 456 | (89) | 184 | 404 | (88) | 189 | 310 | (87) |
| 12 | 123 | 474 | (106) | 122 | 304 | (106) | 128 | 259 | (104) |
| 13 | 75 | 618 | (259) | 69 | 643 | (270) | 74 | 482 | (260) |
| 14 | 36 | 554 | (226) | 38 | 216 | (218) | 37 | 503 | (220) |
| 15 | 13 | 606 | (291) | 12 | 542 | (321) | 14 | 185 | (285) |
| ^a^ Given the missing data assumptions (Supplementary Table 2) complete cost data was obtained for 1556 (95%) of the 1643 men randomized into the ProtecT study | | | | | | | | | |
| ^b^Costs were adjusted for study centre, age at baseline, Gleason score (2-6, 7, 8-10) and PSA at baseline. | | | | | | | | | |

**Supplementary Table 4: Mean adjusted QALYs per year, by arm**

| **Year** | **Active monitoring** | | | **Radiotherapy** | | | **Radical prostatectomy** | | |
| --- | --- | --- | --- | --- | --- | --- | --- | --- | --- |
|  | **N^a^** | **Total QALYs^b^** | **(SE)** | **N^a^** | **Total QALYs^b^** | **(SE)** | **N^a^** | **Total QALYs^b^** | **(SE)** |
| 1 | 377 | 0.88 | (0.01) | 379 | 0.88 | (0.01) | 376 | 0.89 | (0.01) |
| 2 | 377 | 0.88 | (0.01) | 379 | 0.88 | (0.01) | 376 | 0.90 | (0.01) |
| 3 | 377 | 0.88 | (0.01) | 379 | 0.87 | (0.01) | 376 | 0.89 | (0.01) |
| 4 | 377 | 0.87 | (0.01) | 379 | 0.87 | (0.01) | 376 | 0.86 | (0.01) |
| 5 | 377 | 0.86 | (0.01) | 379 | 0.86 | (0.01) | 376 | 0.85 | (0.01) |
| 6 | 377 | 0.85 | (0.01) | 379 | 0.84 | (0.01) | 376 | 0.84 | (0.01) |
| 7 | 369 | 0.83 | (0.01) | 372 | 0.83 | (0.01) | 367 | 0.82 | (0.01) |
| 8 | 313 | 0.82 | (0.01) | 306 | 0.82 | (0.01) | 297 | 0.80 | (0.01) |
| 9 | 238 | 0.81 | (0.02) | 248 | 0.80 | (0.02) | 231 | 0.80 | (0.02) |
| 10 | 172 | 0.77 | (0.02) | 186 | 0.77 | (0.02) | 170 | 0.77 | (0.02) |
| 11 | 104 | 0.77 | (0.03) | 130 | 0.76 | (0.03) | 111 | 0.72 | (0.03) |
| 12 | 69 | 0.78 | (0.04) | 83 | 0.71 | (0.04) | 72 | 0.71 | (0.04) |
| 13 | 31 | 0.83 | (0.05) | 46 | 0.74 | (0.04) | 34 | 0.72 | (0.05) |
| 14 | 15 | 0.87 | (0.08) | 24 | 0.75 | (0.06) | 16 | 0.72 | (0.07) |
| a Given the missing data assumptions (Supplementary Table 2) complete QALY data was obtained for 1132 (69%) of the 1643 men randomized into the ProtecT study | | | | | | | | | |
| **^b^**QALYs were adjusted for study centre, age at baseline, Gleason score (2-6, 7, 8-10),PSA and utility at baseline. | | | | | | | | | |

**Supplementary Table 5: Results of the one-way and scenario sensitivity analyses**

| **Allocation arm** | **N^a^** | **Adjusted costs^b^(£)** | **Adjusted^b^QALYs** | **Comparison** | **Incremental cost (£) (95%CI^c^)** | **Incremental QALY (95%CI^c^)** | **ICER^d^(£/QALY)** | **Incremental NMB (£) at £20000/QALY (95% CI)** |
| --- | --- | --- | --- | --- | --- | --- | --- | --- |
|  |  | **Mean** | **Mean** |  |  |  |  |  |
|  |  | **(95% CI)** | **(95% CI)** |  |  |  |  |  |
| ***Sensitivity analysis 1: Imputed QALYs assumed to be 10% lower*** | | | | | | | | |
| Active monitoring (AM) | 370 | 5,913 | 6.957 |  |  |  |  |  |
|  |  | (5,494 to 6,332) | (6.780 to 7.134) |  |  |  |  |  |
| Radiotherapy (RT) | 364 | 7,361 | 7.073 | RT vs. AM | 1,448 | 0.117 | 12,397 | 888 |
|  |  | (6,938 to 7,783) | (6.895 to 7.252) |  | (771 to 2,065) | (-0.130 to 0.375) |  | (-4,177 to 5,953) |
| Radical prostatectomy (RP) | 367 | 7,519 | 6.889 | RP vs. RT | 159 | -0.184 | RT dominates^e^ RP | -3,838 |
|  |  | (7,099 to 7,940) | (6.712 to 7.067) |  | (-417 to 727) | (-0.433 to 0.068) |  | (-8,910 to 1,234) |
| ***Sensitivity analysis 2: No imputed QALY data*** | | | | | | | | |
| Active monitoring (AM) | 284 | 5,861 | 6.808 |  |  |  |  |  |
|  |  | (5,366 to 6,355) | (6.613 to 7.002) |  |  |  |  |  |
| Radiotherapy (RT) | 273 | 7,384 | 6.863 | RT vs. AM | 1,523.34 | 0.055 | 27,673 | -422 |
|  |  | (6,879 to 7,889) | (6.664 to 7.061) |  | (709 to 2,225) | (-0.242 to 0.341) |  | (-6,028 to 5,183) |
| Radical prostatectomy (RP) | 276 | 7,478 | 6.751 | RP vs. RT | 94.32 | -0.112 | RT dominates RP | -2,327 |
|  |  | (6,975 to 7,981) | (6.553 to 6.949) |  | (-566 to 765) | (-0.391 to 0.164) |  | (-7,982 to 3,327) |
| ***Sensitivity analysis 3: Including only participants recruited following the introduction of primary care data collection*** | | | | | | | | |
| Active monitoring (AM) | 274 | 5,672 | 6.417 |  |  |  |  |  |
|  |  | (5,169 to 6,175) | (6.248 to 6.586) |  |  |  |  |  |
| Radiotherapy (RT) | 244 | 7,122 | 6.471 | RT vs. AM | 1,450 | 0.054 | 27,009 | -376 |
|  |  | (6,588 to 7,656) | (6.292 to 6.650) |  | (694 to 2,143) | (-0.189 to 0.303) |  | (-5,351 to 4,599) |
| Radical prostatectomy (RP) | 269 | 7,691 | 6.363 | RP vs. RT | 569 | -0.107 | RT dominates RP | -2,714 |
|  |  | (7,182 to 8,199) | (6.193 to 6.534) |  | (-122 to 1,277) | (-0.341 to 0.119) |  | (-7,707 to 2,279) |

**Supplementary Table 5: Results of the one-way and scenario sensitivity analyses (continued)**

| **Allocation arm** | **N^a^** | **Adjusted cost^sb^(£)** | **Adjusted^b^QALYs** | **Comparison** | | **Incremental cost (£) (95%CI^c^)** | **Incremental QALY (95%CI^c^)** | **ICER^d^(£/QALY)** | | | **Incremental NMB (£) at £20000/QALY (95% CI)** |
| --- | --- | --- | --- | --- | --- | --- | --- | --- | --- | --- | --- |
|  |  | **Mean** | **Mean** |  |  |  |  |  |  |  |  |
|  |  | **(95% CI)** | **(95% CI)** |  |  |  |  |  |  |  |  |
| ***Sensitivity analysis 4a: Only including data from the first 6 years*** | | | | | | | | | | | |
| Active monitoring (AM) | 393 | 4,464 | 4.798 |  |  | |  | |  |  | |
|  |  | (4,128 to 4,801) | (4.724 to 4.872) |  |  | |  | |  |  | |
| Radiotherapy (RT) | 383 | 6,481 | 4.835 | RT vs. AM | 2,017 | | 0.037 | | 54,283 | -1,274 | |
|  |  | (6,140 to 6,822) | (4.760 to 4.910) |  | (1,479 to 2,461) | | (-0.073 to 0.144) | |  | (-3,472 to 924) | |
| Radical prostatectomy (RP) | 388 | 6,769 | 4.823 | RP vs. RT | 288 | | -0.012 | | RT dominates RP | -524 | |
|  |  | (6,431 to 7,108) | (4.749 to 4.897) |  | (-141 to 720) | | (-0.108 to 0.088) | |  | (-2,728 to 1,679) | |
| ***Sensitivity analysis 4b: Only including data from the first 6 years, controlling for the information appointment date*** | | | | | | | | | | | |
| Active monitoring (AM) | 393 | 4,466 | 4.797 |  |  | |  | |  |  | |
|  |  | (4,131 to 4,801) | (4.724 to 4.871) |  |  | |  | |  |  | |
| Radiotherapy (RT) | 383 | 6,488 | 4.834 | RT vs. AM | 2,022 | | 0.036 | | 55,860 | -1,298 | |
|  |  | (6,148 to 6,828) | (4.759 to 4.908) |  | (1,469 to 2,465) | | (-0.065 to 0.144) | |  | (-3,487 to 891) | |
| Radical prostatectomy (RP) | 388 | 6,761 | 4.824 | RP vs. RT | 273 | | -0.009 | | RT dominates RP | -454 | |
|  |  | (6,424 to 7,099) | (4.750 to 4.899) |  | (-154 to 712) | | (-0.108 to 0.093) | |  | (-2,649 to 1,741) | |
| ***Sensitivity analysis 5: Excluding men recruited during the feasibility period*** | | | | | | | | | | | |
| Active monitoring (AM) | 350 | 5,854 | 6.816 |  |  | |  | |  |  | |
|  |  | (5,423 to 6,285) | (6.644 - 6.987) |  |  | |  | |  |  | |
| Radiotherapy (RT) | 334 | 7,285 | 6.887 | RT vs. AM | 1,431 | | 0.071 | | 20,195 | -14 | |
|  |  | (6,844 to 7,727) | (6.711 - 7.062) |  | (793 to 2,056) | | (-0.169 to 0.317) | |  | (-4,943 to 4,915) | |
| Radical prostatectomy (RP) | 347 | 7,568 | 6.766 | RP vs. RT | 282 | | -0.120 | | RT dominates RP | -2,692 | |
|  |  | (7,134 to 8,001) | (6.594 - 6.938) |  | (324 to 839) | | (-0.367 to 0.116) | |  | (-7,629 to 2,246) | |

**Supplementary Table 5: Results of the one-way and scenario sensitivity analyses(continued)**

| **Allocation arm** | **N^a^** | **Adjusted costs^b^(£)** | **Adjusted^b^QALYs** | **Comparison** | **Incremental cost (£) (95%CI^c^)** | | **Incremental QALY (95%CI^c^)** | | **ICER^d^(£/QALY)** | | **Incremental NMB (£) at £20000/QALY (95% CI)** |
| --- | --- | --- | --- | --- | --- | --- | --- | --- | --- | --- | --- |
|  |  | **Mean** | **Mean** |  |  |  |  |  |  |  |  |
|  |  | **(95% CI)** | **(95% CI)** |  |  |  |  |  |  |  |  |
| ***Sensitivity analysis 6: Discount rates of 1.5% for costs and QALYs*** | | | | | | | | | | | |
| Active monitoring (AM) | 370 | 6,345 | 7.560 |  |  |  | |  | |  | |
|  |  | (5,889 to 6,801) | (7.356 to 7.765) |  |  |  | |  | |  | |
| Radiotherapy (RT) | 364 | 7,618 | 7.696 | RT vs. AM | 1,273 | 0.136 | | 9,358 | | 1,447 | |
|  |  | (7,158 to 8,077) | (7.490 to 7.903) |  | (563 to 1,933) | (-0.154 to 0.432) | |  |  | (-4,388 to 7,283) | |
| Radical prostatectomy (RP) | 367 | 7,745 | 7.476 | RP vs. RT | 127 | -0.221 | | RT dominates RP | | -4,541 | |
|  |  | (7,287 to 8,202) | (7.270 to 7.681) |  | (-471 to 738) | (-0.515 to 0.069) | |  |  | (-10,383 to 1,302) | |
| ***Sensitivity analysis 7: Day cases costed as outpatients*** | | | | | | | | | | | |
| Active monitoring (AM) | 370 | 5,774 | 6.976 |  |  |  | |  | |  | |
|  |  | (5,375 to 6,173) | (6.798 to 7.154) |  |  |  | |  | |  | |
| Radiotherapy (RT) | 364 | 7,247 | 7.094 | RT vs. AM | 1,473 | 0.118 | | 12,530.66 | | 878 | |
|  |  | (6,845 to 7,650) | (6.915 to 7.273) |  | (810 to 2,059) | (-0.153 to 0.368) | |  |  | (-4,204 to 5,961) | |
| Radical prostatectomy (RP) | 367 | 7,375 | 6.909 | RP vs. RT | 128 | -0.185 | | RT dominates RP | | -3,830 | |
|  |  | (6,974 to 7,776) | (6.730 to 7.087) |  | (-422 to 647) | (-0.430 to 0.068) | |  |  | (-8,919 to 1,260) | |
| ***Sensitivity analysis 8: Excluding TURPs*** | | | | | | | | | | | |
| Active monitoring (AM) | 370 | 5,814 | 6.976 |  |  |  | |  | |  | |
|  |  | (5,396 to 6,232) | (6.798 to 7.154) |  |  |  | |  | |  | |
| Radiotherapy (RT) | 364 | 7,323 | 7.094 | RT vs. AM | 1,509 | 0.118 | | 12,829.16 | | 843 | |
|  |  | (6,901 to 7,745) | (6.915 to 7.273) |  | (834 to 2,106) | (-0.143 to 0.374) | |  |  | (-4,243 to 5,930) | |
| Radical prostatectomy (RP) | 367 | 7,520 | 6.909 | RP vs. RT | 197 | -0.185 | | RT dominates RP | | -3,899 | |
|  |  | (7,100 to 7,940) | (6.730 to 7.087) |  | (-373 to 767) | (-0.445 to 0.055) | |  |  | (-8,992 to 1,195) | |

**Supplementary Table 5: Results of the one-way and scenario sensitivity analyses(continued)**

| **Allocation arm** | **N^a^** | **Adjusted costs^b^(£)** | **Adjusted^b^QALYs** | **Comparison** | **Incremental cost (£) (95%CI^c^)** | | **Incremental QALY (95%CI^c^)** | | **ICER^d^(£/QALY)** | | **Incremental NMB (£) at £20000/QALY (95% CI)** |
| --- | --- | --- | --- | --- | --- | --- | --- | --- | --- | --- | --- |
|  |  | **Mean** | **Mean** |  |  |  |  |  |  |  |  |
|  |  | **(95% CI)** | **(95% CI)** |  |  |  |  |  |  |  |  |
| ***Sensitivity analysis 9: Excluding annual/research follow-ups*** | | | | | | | | | | | |
| Active monitoring (AM) | 370 | 5,893 | 6.976 |  |  |  | |  | |  | |
|  |  | (5,475 to 6,312) | (6.798 to 7.154) |  |  |  | |  | |  | |
| Radiotherapy (RT) | 364 | 7,330 | 7.094 | RT vs. AM | 1,436 | 0.118 | | 12,215.11 | | 915 | |
|  |  | (6,908 to 7,752) | (6.915 to 7.273) |  | (790 to 2,046) | (-0.137 to 0.370) | |  |  | (-4,170 to 6,001) | |
| Radical prostatectomy (RP) | 367 | 7,495 | 6.909 | RP vs. RT | 165 | -0.185 | | RT dominates RP | | -3,867 | |
|  |  | (7,075 to 7,915) | (6.730 to 7.087) |  | (-402 to 745) | (-0.439 to 0.059) | |  |  | (-8,960 to 1,225) | |
|  |  |  |  |  |  |  | |  | |  | |
| ***Sensitivity analysis 10: Using an alternative number of fractions (n=20) for radiotherapy delivery*** | | | | | | | | | | | |
| Active monitoring (AM) | 370 | 5,582 | 6.976 |  |  |  | |  | |  | |
|  |  | (5,171 to 5,992) | (6.798 to 7.154) |  |  |  | |  | |  | |
| Radiotherapy (RT) | 364 | 5,974 | 7.094 | RT vs. AM | 393 | 0.118 | | 3,339.44 | | 1,959 | |
|  |  | (5,560 to 6,388) | (6.915 to 7.273) |  | (-212 to 963) | (-0.136 to 0.369) | |  |  | (-3,123 to 7,041) | |
| Radical prostatectomy (RP) | 367 | 7,385 | 6.909 | RP vs. RT | 1,411 | -0.185 | | RT dominates RP | | -5112 | |
|  |  | (6,972 to 7,797) | (6.730 to 7.087) |  | (847 to 1,960) | (-0.433 to 0.064) | |  |  | (-10,201 to -24) | |
|  |  |  |  |  |  |  | |  | |  | |
| ***Sensitivity analysis 11: Including data from the procedures section of the annual follow-up schedule*** | | | | | | | | | | | |
| Active monitoring (AM) | 370 | 5,946 | 6.976 |  |  |  | |  | |  | |
|  |  | (5,526 to 6,367) | (6.798 to 7.154) |  |  |  | |  | |  | |
| Radiotherapy (RT) | 364 | 7,391 | 7.094 | RT vs. AM | 1,445 | 0.118 | | 12,284.32 | | 907 | |
|  |  | (6,967 to 7,815) | (6.915 to 7.273) |  | (798 to 2,035) | (-0.142 to 0.369) | |  |  | (-4,178 to 5,993) | |
| Radical prostatectomy (RP) | 367 | 7,552 | 6.909 | RP vs. RT | 161 | -0.185 | | RT dominates RP | | -3,863 | |
|  |  | (7,130 to 7,975) | (6.730 to 7.087) |  | (-413 to 744) | (-0.429 to 0.069) | |  |  | (-8,955 to 1,229) | |

**Supplementary Table 5: Results of the one-way and scenario sensitivity analyses(continued)**

| **Allocation arm** | | **N^a^** | | **Adjusted costs^b^(£)** | | **Adjusted^b^QALYs** | | **Comparison** | | **Incremental cost (£) (95%CI^c^)** | | **Incremental QALY (95%CI^c^)** | | **ICER^d^(£/QALY)** | | | **Incremental NMB (£) at £20000/QALY (95% CI)** | |  |
| --- | --- | --- | --- | --- | --- | --- | --- | --- | --- | --- | --- | --- | --- | --- | --- | --- | --- | --- | --- |
|  |  |  |  | **Mean** | | **Mean** | |  |  |  |  |  |  |  |  |  |  |  |  |
|  |  |  |  | **(95% CI)** | | **(95% CI)** | |  |  |  |  |  |  |  |  |  |  |  |  |
| ***Scenario analysis 1: Outpatient TWOCs costed as day cases, outpatient, non-consultant led visits for PSAs, inpatient procedures costed at highest CC split, annual/research follow-ups costed at full cost, protocol radiotherapy preparation includes technical support*** | | | | | | | | | | | | | | | | | | | |
| Active monitoring (AM) | 370 | | 8,525 | | 6.976 | |  | |  | |  | |  | | |  | | | |
|  |  |  | (7,905 to 9,145) | | (6.798 to 7.154) | |  | |  | |  | |  | | |  | | | |
| Radiotherapy (RT) | 364 | | 9,343 | | 7.094 | | RT vs. AM | | 818 | | 0.117 | | 6,967.01 | | | 1,531 | | | |
|  |  |  | (8,718 to 9,969) | | (6.914 to 7.273) | |  |  | (-118 to 1,670) | | (-0.122 to 0.368) | |  |  |  | (-3,569 to 6,630) | | | |
| Radical prostatectomy (RP) | 367 | | 9,905 | | 6.909 | | RP vs. RT | | 561 | | -0.185 | | RT dominates RP | | | -4,263 | | | |
|  |  |  | (9,282 to 10,528) | | (6.730 to 7.087) | |  |  | (-296 to 1,405) | | (-0.437 to 0.063) | |  |  |  | (-9,369 to 843) | | | |
| ***Scenario analysis 2: Current treatment costs: Protocol radiotherapy costed as IMRT and radical prostatectomies costed as robotic*** | | | | | | | | | | | | | | | | | |  |  |
| Active monitoring (AM) | 370 | | 7,097 | | 6.976 | |  | |  | |  | |  | |  | | |  |  |
|  |  |  | (6,615 to 7,578) | | (6.798 to 7.154) | |  | |  | |  | |  | |  | | |  |  |
| Radical prostatectomy (RP) | 367 | | 9,552 | | 6.909 | | RP vs. AM | | 2,455 | | -0.068 | | AM dominates RP | | -3,805 | | |  |  |
|  |  |  | (9,068 to 10,035) | | (6.730 to 7.087) | |  |  | (1,732 to 3,126) | | (-0.327 to 0.195) | |  |  | (-8,889 to 1,279) | | |  |  |
| Radiotherapy (RT) | 364 | | 10,487 | | 7.094 | | RT vs. AM | | 3,391 | | 0.118 | | 28,833.67 | | -1,039 | | |  |  |
|  |  |  | (10,002 to 10,973) | | (6.915 to 7.273) | |  |  | (2,608 to 4,112) | | (-0.138 to 0.367) | |  |  | (-6,135 to 4,057) | | |  |  |
| ^a^Including only participants for whom we have complete cost and QALY information given the assumptions in supplementary table 2 | | | | | | | | | | | | | | | | | |  |  |
| ^b^Adjusted for the minimisation variables of the randomization process: study centre, age at baseline, Gleason score (2-6, 7, 8-10) and PSA at baseline. QALYs were also adjusted for baseline utility | | | | | | | | | | | | | | | | | |  |  |
| ^c^Bias-corrected and accelerated confidence interval based on 5000 bootstrap replications | | | | | | | | | | | | | | | | | |  |  |
| ^d^ICERs cannot be estimated directly from the incremental costs and QALYs due to rounding | | | | | | | | | | | | | | | | | |  |  |
| ^e^ dominates means that the treatment is less costly and more effective than the other treatment | | | | | | | | | | | | | | | | | |  |  |

**Supplementary Table 6: Baseline characteristics of those included and excluded from the SUR analysis by arm**

|  | **Active monitoring** | | | | **Radiotherapy** | | | | **Radical Prostatectomy** | | | | |
| --- | --- | --- | --- | --- | --- | --- | --- | --- | --- | --- | --- | --- | --- |
|  | **Included** | | **Excluded** | | **Included** | | **Excluded** | | **Included** | | **Excluded** | |  |
|  | **n** | **Mean (SD)** | **n** | **Mean (SD)** | **n** | **Mean (SD)** | **n** | **Mean (SD** | **n** | **Mean (SD** | **n** | **Mean (SD** |  |
| **Age** | 370 | 62 (5) | 175 | 62 (5) | 364 | 62 (5) | 181 | 62 (5) | 367 | 62 (5) | 186 | 62 (5) |  |
| **PSA μg/L** | 370 | 5.7 (3.0) | 175 | 5.9 (3.2) | 364 | 5.8 (3.0) | 181 | 5.7 (3.0) | 367 | 5.7 (2.8) | 186 | 6.1 (3.6) |  |
| **Utility** | 370 | 0.88 (0.18) | 104 | 0.86 (0.21) | 364 | 0.90 (0.15) | 94 | 0.88 (0.19) | 367 | 0.90 (0.17) | 114 | 0.84 (0.19) |  |
|  |  | **n (%)** |  | **n (%)** |  | **n (%)** |  | **n (%)** |  | **n (%)** |  | **n (%)** |  |
| **Gleason score** |  |  |  |  |  |  |  |  |  |  |  |  |  |
| **2-6** |  | 277 (75) |  | 144 (82) |  | 279 (77) |  | 144 (80) |  | 274 (75) |  | 149 (80) |  |
| **7** |  | 85 (23) |  | 26 (15) |  | 75 (21) |  | 33 (18) |  | 90 (25) |  | 30 (16) |  |
| **8-10** |  | 8 (2) |  | 5 (3) |  | 10 (3) |  | 4 (2) |  | 3(1) |  | 7 (4) |  |

References

1. NICE. Guide to the methods of technology appraisal 2013. <https://www.nice.org.uk/process/pmg9/chapter/the-reference-case#framework-for-estimating-clinical-and-cost-effectiveness.Accessed> April 18,2018
2. Gov.UK. NHS reference costs 2014 to 2015. <https://www.gov.uk/government/publications/nhs-reference-costs-2014-to-2015>. Accessed April 18,2018
3. Curtis LA, Burns A. Unit Costs of Health and Social Care 2015.<http://www.pssru.ac.uk/project-pages/unit-costs/unit-costs-2015/>. Accessed April 18,2018
4. (NHSBSA) NBSA. Dictionary of Medicines and Devices. <https://www.nhsbsa.nhs.uk/pharmacies-gp-practices-and-appliance-contractors/dictionary-medicines-and-devices-dmd>. Accessed April 18,2018
5. NHS Digital. HRG4+ 2014/15 Reference Cost Grouper 2014/2015. <http://webarchive.nationalarchives.gov.uk/20171012004847/http://content.digital.nhs.uk/article/6226/HRG4-201415-Reference-Cost-Grouper> Accessed April 18,2018
